# Soybean yield estimation and lodging discrimination based on lightweight UAV and point cloud deep learning

**DOI:** 10.1016/j.plaphe.2025.100028

**Published:** 2025-03-20

**Authors:** Longyu Zhou, Dezhi Han, Guangyao Sun, Yaling Liu, Xiaofei Yan, Hongchang Jia, Long Yan, Puyu Feng, Yinghui Li, Lijuan Qiu, Yuntao Ma

**Affiliations:** a College of Land Science and Technology, China Agricultural University, Beijing, 100193, China; b Heihe Branch of Heilongjiang Academy of Agricultural Sciences, Heihe, China; c College of Information and Electrical Engineering, China Agricultural University, Beijing 100193, China; d Inner Mongolia Pratacultural Technology Innovation Center Co. Ltd, Inner Mongolia, China; e Institute of Cereal and Oil Crops, Hebei Academy of Agricultural and Forestry Sciences, Shijiazhuang, Hebei, 050035, China; f State Key Laboratory of Crop Gene Resources and Breeding, Institute of Crop Science, Chinese Academy of Agricultural Sciences, Beijing, 100081, China

**Keywords:** Remote sensing, 3D reconstruction, Point cloud, Multi-task learning, Digital image

## Abstract

The unmanned aerial vehicle (UAV) platform has emerged as a powerful tool in soybean (Glycine max (L.) Merr.) breeding phenotype research due to its high throughput and adaptability. However, previous studies have predominantly relied on statistical features like vegetation indices and textures, overlooking the crucial structural information embedded in the data. Feature fusion has often been confined to a one-dimensional exponential form, which can decouple spatial and spectral information and neglect their interactions at the data level. In this study, we leverage our team's cross-circling oblique (CCO) route photography and Structure-from-Motion with Multi-View Stereo (SfM-MVS) techniques to reconstruct the three-dimensional (3D) structure of soybean canopies. Newly point cloud deep learning models SoyNet and SoyNet-Res were further created with two novel data-level fusion that integrate spatial structure and color information. Our results reveal that incorporating RGB color and vegetation index (VI) spectral information with spatial structure information, leads to a significant reduction in root mean square error (RMSE) for yield estimation (22.55 ​kg ​ha^−1^) and an improvement in F1-score for five-class lodging discrimination (0.06) at S7 growth stage. The SoyNet-Res model employing multi-task learning exhibits better accuracy in both yield estimation (RMSE: 349.45 ​kg ​ha^−1^) when compared to the H2O-AutoML. Furthermore, our findings indicate that multi-task deep learning outperforms single-task learning in lodging discrimination, achieving an accuracy top-2 of 0.87 and accuracy top-3 of 0.97 for five-class. In conclusion, the point cloud deep learning method exhibits tremendous potential in learning multi-phenotype tasks, laying the foundation for optimizing soybean breeding programs.

## 1 Introduction

Soybean (Glycine max (L.) Merr.) is a globally important crop, serving as a key ingredient in food, feed, and industrial products [1]. To meet the growing demands of increasing population and industrial production, enhancing soybean yield is an important task for breeding programs [2]. Therefore, accurate evaluation of phenotypic traits in breeding lines is crucial for efficiently identifying superior and high-yielding varieties [[3], [4], [5], [6]].

Crop breeding processes typically involve the examination of hundreds or even thousands of breeding materials. However, traditional field surveys are time-consuming, labor-intensive, and costly. They not only have limitations in investigating various traits, but also fail to identify most of them in a timely manner. In recent years, significant advancements in unmanned aerial vehicles (UAV) proximity sensing platforms and information technology have provided effective means for monitoring crop phenotypes and facilitating breeding decisions [5,6]. These platforms are equipped with various sensors, including multispectral, hyperspectral and LiDAR sensors, which enable the extraction of phenotypic features such as VIs, canopy cover (CC), texture [[6], [7], [8]]. These features have been utilized for estimating biomass [9,10], yield [11], nitrogen content [12], and chlorophyll content [13], as well as for early diagnosis of nutritional elements [14] and disease identification at the plot scale [15].

Compared to 2D data suffering from limited resolution and occlusion problems, 3D data offer more comprehensive information of crop structural [16,17]. The methods for acquiring with 3D crop high-precision data are primarily categorized into Light Detection and Ranging (LiDAR) and photogrammetry. LiDAR has been successfully employed in crop phenotype monitoring. However, its application in agriculture is constrained by its high cost and weight [18,19]. Photogrammetry, equipped with consumer-grade high-resolution RGB cameras, effectively generates dense point clouds and color information from sparse point clouds through the fusion of Structure from Motion (SfM) and Multi-View Stereo (MVS) techniques [20,21]. However, oblique photography with straight lines is limited by terrain constraints and crop occlusion, resulting in an insufficient number of viewpoints in complex field environments [17,22]. In contrast, the UAV cross-circling oblique (CCO) imaging [19] enables adjustable flight heights and shooting angles based on specific crop conditions. This approach economically and efficiently captures more comprehensive land surface information as well as higher quality point cloud data [17,22,23].

For 3D data, the extraction of crop attribute characteristics in the spatial structure is particularly effective. Data in 3D form mainly includes point cloud, polygon grid and 3D digital model. Point cloud data is the real sampling of crop canopy, which is easier to process and use than polygonal grid data [17,22]. Compared with the virtual construction of 3D data model, point cloud data is real and reliable. Utilizing the Digital Elevation Model (DEM), the crop canopy model can be derived to extract plant height parameters [6,24]. Crop structural features can be obtained from 3D point clouds, such as 3D voxel index (3DVI) and 3D profile index (3DPI) [25], along with the percentage for different plant height [26], etc. Statistical based index features facilitate phenotypic parameter estimation, but these easy-to-understand shallow features, especially those in point clouds, may overlook deeper structural information and more abstract features within the data [27,28].

Another simpler and more efficient method for feature extraction is to directly extract the features of the point cloud using deep learning techniques. In comparison to the methods that rely on 2D data and employ statistics or Convolutional Neural Networks (CNNs) for phenotypic feature extraction, the application of point cloud deep learning for phenotypic estimation remains understudied. Oehmcke [28] developed a regression model based on LiDAR data, highlighting the untapped potential of point cloud regression deep learning. Unlike single-task learning models that target specific predictions, multi-task learning models simultaneously learn multiple tasks by sharing model parameters and capturing potential correlation features between tasks. This approach effectively enhances both the efficiency and accuracy of the learning process [[29], [30], [31]]. Therefore, it is imperative to develop an efficient point cloud deep learning method capable of fully extracting structural information from crops and directly employing it for multi-task phenotype monitoring.

In previous phenotypic research on multi-modal data fusion, UAVs have primarily been equipped with multiple sensors from various sources to acquire data and extract features. Subsequently, these features are then fused either at the feature level or the decision level [7,17,31,32]. However, there is a scarcity of studies that directly extract multi-modal data at the raw data level. A deep learning model was proposed here, capable of simultaneously inputting and processing both spatial structure information and color information from point clouds, thereby achieving data-level fusion of multi-modal data: (1) The CCO photography technology was combined with the SfM-MVS algorithm to generate high-precision and cost-effective 3D point clouds for different soybean varieties. (2) Multi-task learning models (SoyNet and SoyNet-RES) tailored for current mainstream phenotype estimation tasks were proposed for regression tasks and multi-classification tasks. Spatial and spectral information were fused at the data level, to estimate soybean yield and discriminate lodging. (3) The stability of different structures and the accuracy of time series in point cloud deep learning models are compared, to evaluate the estimation accuracy, spatial robustness, and adaptability of single-task and multi-task learning strategies.

## 2 Material

### 2.1 Experimental site and setup

**Fig. 1:**
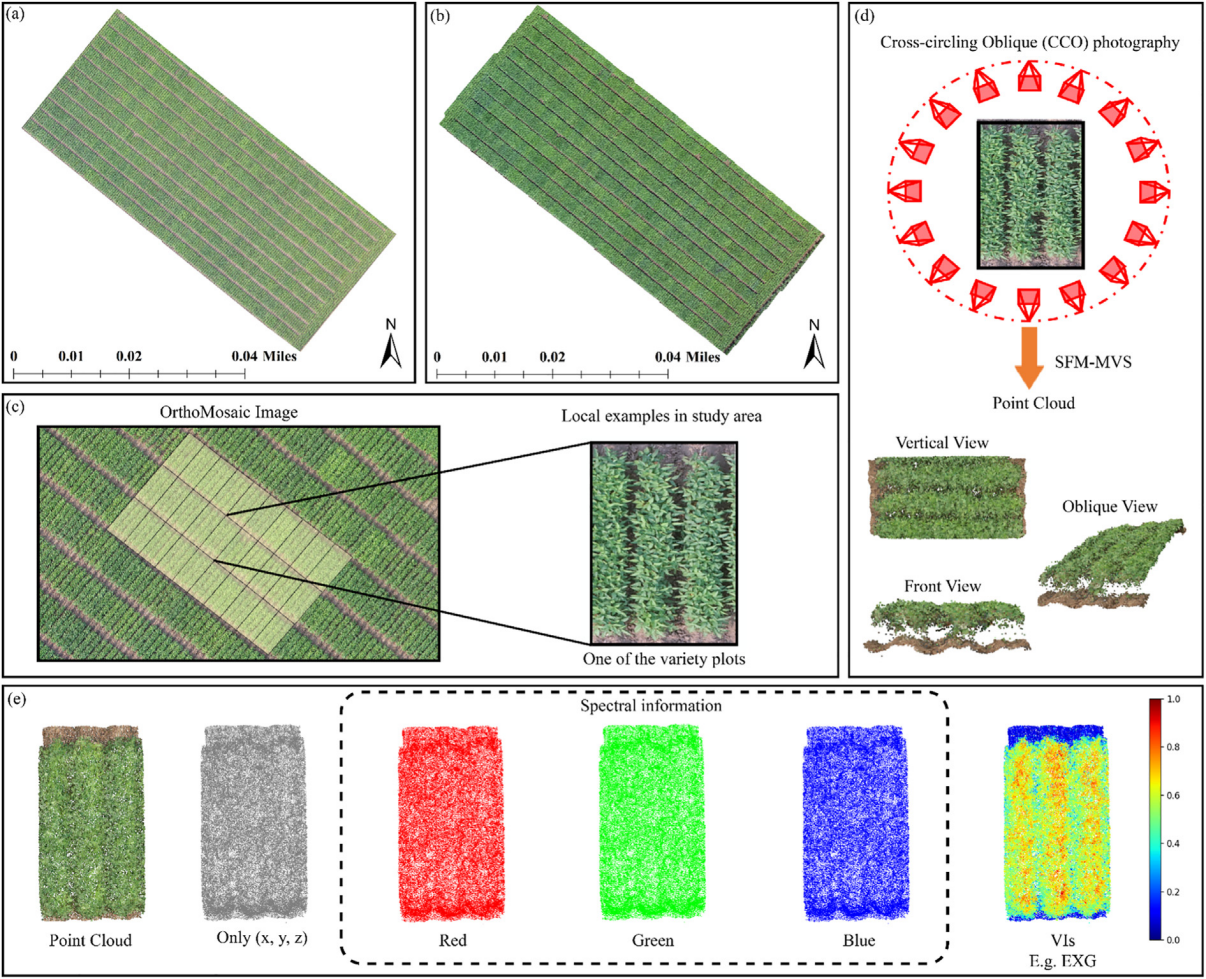
The geographical location of the experimental field and schematic diagram of the field design are as follows: (a) An orthomosaic image was generated by stitching RGB images of soybean taken on July 10, 2023; (b) An orthomosaic image was generated by stitching RGB images of soybean taken on July 20, 2023; (c) After segmenting the orthomosaic image, an example cell was selected; (d) The CCO route method was utilized to obtain high-precision point cloud information and was visualized a single cell from top view, front view, and oblique view perspectives; (e) Spectral information decomposition of the point cloud includes: cropped point cloud, point cloud with only spatial information (x, y, z), red band (R), green band (G), blue band information(B), and VIs calculated using red, green, and blue bands. The figure demonstrated the EXG index as an example.

The experimental site was situated in Heihe City, Heilongjiang Province, China (47° 42' - 51° 03′ N, 124° 45' - 129° 18′E). Heihe City was positioned at the eastern terminus of the Greater Khingan Mountains and the northern section of the Lesser Khingan Mountains. It experienced a cold temperate continental monsoon climate characterized by elevated temperatures and winds during spring, as well as heavy rainfall in summer. The cultivated crop (Fig. 1ab) in the experimental site was soybean, and the ridge length was 3.1 ​m. The width was 0.6 ​m, and the area was about 1.9 ​m^2^. The combination of three ridges was a plot area of one variety (Fig. 1c). The spacing between ridges was about 0.3 ​m, and the plot area was about 5.9 ​m^2^.

In the study area, 186 soybean varieties were planted, with a total of 558 plots, in which the low (D1), medium (D2) and high (D3) planting densities were 3.0 ​× ​105 seeds ha^−1^, 3.5 ​× ​105 seeds ha^−1^ and 4.2 ​× ​105 seeds ha^−1^, respectively. All soybean varieties were sowed on May 14, 2023, followed by planted on May 30, 2023, and harvested on October 19, 2023. Field management was conducted in accordance with conventional specifications.

### 2.2 Data acquisition

#### 2.2.1 Field data collection

The canopy height, bottom pod height, main stem nodes number, branch number, pods per plant, grains per plant, 100-seed weight, grain yield and lodging grade of soybean were quantified and assessed across 558 plot groups. Lodging level was determined through manual visual assessment and classified into five levels: no lodging (level 1), mild lodging (level 3), moderate lodging (level 5), severe lodging (level 7) and complete lodging (level 9). Each plot was harvested, threshed and dried individually, weighed when the soybean moisture content was below 13%. As shown in Table 1, which shows statistics of field.

**Table 1:** Descriptive statistics for field-measured parameters.

Parameters	Mean	Max.	Min.	SD	CV(%)
Canopy height (m)	0.94	1.54	0.53	0.14	14.69
Bottom pod height(m)	0.31	1.06	0.09	0.09	30.43
Main stem nodes	12.24	16.80	7.60	1.42	11.57
Branches	0.14	2.20	0.00	0.31	226.96
Pods per plant	20.64	43.40	9.80	5.37	26.01
Grains per plant	46.41	102.00	15.80	12.05	25.97
100-seed weight(g)	16.69	21.87	7.50	2.32	13.89
Grain yield (kg ha^−1^)	2529.75	3860.00	760.00	467.89	18.50

SD: standard deviation; CV: coefficient of variation.

#### 2.2.2 Methods of UAV data acquisition

The UAV survey was conducted in multiple stages on July 1 (S1), July 10 (S2), July 20 (S3), July 28 (S4), August 14 (S5), September 2 (S6), and September 13 (S7) in 2023. DJI Phantom 4 RTK was used to collect the RGB images with resolution of 4608 ​× ​3456 pixels. UAV employed two flight modes: (1) when operating at an altitude of 15 ​m, it utilized oblique photography with 85 ​% forward and lateral overlap for image capture. The RGB image had a spatial resolution of 0.38 cm/pixel. (2) For acquiring canopy point cloud data with high-precision (Fig. 1d), the UAV flied at a height of 5 ​m and followed a cross-circling oblique (CCO) route [22]. The overlap rate was 50 ​% and the camera tilt Angle was 45°. All UAV image collection was performed during 11:00 a.m. and 14:00 p.m. under clear, windless weather conditions.

#### 2.2.3 Raw data preprocessing

After completing the UAV orthophoto shooting task, the captured data undergo geometric correction and stitching processes with Agisoft Metashape software (Agisoft LLC, St. Petersburg, Russia). This involves image alignment, GCP import, and dense point cloud generation. The oblique photography data was used to generate the digital orthomosaic map (DOM) and digital surface model (DSM). DSM of early bare soil surface in the study area was established. Canopy plant height is obtained by subtracting it from subsequent at different crop growth stage DSMs [7].

The image data collected by the CCO route was processed using Agisoft Metashape software for image alignment, resulting in the generation of a dense point cloud (Fig. 1d) for subsequent modeling purposes. QGIS 3.24 software was utilized to establish a vector layer and segment individual plot (Fig. 1c), enabling the acquisition of RGB orthophoto, plant height, and point cloud data specific to that plot. During the processing of the point cloud data, abnormal and noisy points were initially eliminated, followed by ground area fitting using the Random Sample Consensus (RANSAC) method [33]. RANSAC [33] is an iterative algorithm used to estimate model parameters from a data set containing outliers. The basic concept is to fit the model by randomly selecting a subset of data and identify a set of inner points based on a preset error threshold to determine which data points fit the model. After many iterations, the model with the most internal points is considered the best model. In this study, RANSAC was used to improve the robustness of the model to outliers, ensuring that the selected model accurately reflects the main features of the data. This allowed for separation between ground surfaces and soybean plants within the dataset. Ultimately, high-precision point cloud data pertaining to the soybean canopy were retained.

## 3 Methods

Utilization of point cloud with deep learning network is proposed in this study in conjunction with RGB color information to accurately estimate soybean yield during various growth stages and to enable multi-class discrimination of lodging. The effectiveness of deep learning in phenotype estimation with point cloud is evaluated, and compared with commonly used methods such as VIs and texture feature analysis.

### 3.1 Extraction of canopy spectral, structural and texture information

The UAV orthophoto comprises three bands, namely red (R), green (G) and blue (B). These bands are utilized to establish the VIs presented in Table 2 for yield estimation and lodging discrimination. The high-resolution orthophoto image is employed to create the CIVE index (Table 2.), which facilitated plant and soil segmentation followed by soybean canopy cover extraction [17].

**Table 2:** Extractions of vegetation indices and texture features.

Data type/info	Features	Formulation	References
VIs	Excess red index (EXR)	1.4R-G	[34]
	Excess green index (EXG)	2G-R-B	[35]
	Excess green minus excess red (EXGR)	EXG-EXR	[36]
	Modify green-Red Vegetation index (MGRVI)	(G^2^-R^2^)/(G^2^+R^2^)	[37]
	Red green ratio index (RGRI)	R/H	[38]
	Plant Pigment Tatio (PPRb)	(G-B)/(G ​+ ​B)	[39]
	Visible atmospherically resistant index (VARI)	(G-R)/(G ​+ ​R-B)	[40]
	Woebbecke index (WI)	(G-B)/(R-G)	[35]
	Vegetativen (VEG)	G/(R^0.667^∗B^0.333^)	[41]
	Color index of vegetation extraction (CIVE)	0.441R-0.811G+0.385B ​+ ​18.79	[42]
	Gray-level co-occurrence matrix (GLCM)	HO, CO, DI, EN, SE, CO	[43]
DSM	Canopy Height (m)	DSM ​− ​DEM	
Point cloud	Quantile of point height	95 ​%	[44]
	Statistical height of point	Mean, Max	[44]
	Convex hull area	/	[44]
	Density percentage	0.5, 0.4, 0.3, 0.2, 0.1, 0.05	[44]

In the field of UAV-based high-throughput plant phenotyping, texture information has been demonstrated to significantly contribute to yield and lodging monitoring [45]. Therefore, the gray level co-occurrence matrix (GLCM) [43] is employed for extracting RGB texture feature information in this study. The RGB image is then converted into a grayscale image allowing us for the extraction of homogeneity (HO), contrast (CO), dissimilarity (DI), entropy (EN), second moment (SE), and correlation (CO). The extracted features are presented in Table 2.

The plant height of soybean at different growth stages is also a crucial information. In this study, high-resolution RGB images are acquired through UAV oblique photography and stitched together to construct a Digital Elevation Model (DEM). The DSM of soybean during various growth stages is subtracted from the DEM of the early bare soil surface at the pixel level to derive the average height of the soybean canopy within the region. The average height, 95 ​% quantile height and, the maximum height of the point cloud is extracted as a supplement to the canopy height information. Then, the convex hull volume of the point cloud and the percentage information of different voxels are extracted as the canopy structure information [44].

### 3.2 Crop phenotype estimation based on point cloud deep learning

#### 3.2.1 Point cloud data augmentation

To fully integrate the spectral dimension information associated with the point cloud, RGB information is utilized to calculate vegetation indices (EXR, EXG, EXGR, MGRVI, RGRI, PPRb, VARI) as inputs for the model, as illustrated in Fig. 1e. Due to the large number of point clouds within an individual soybean plot, the input network consumes a lot of memory. Therefore, voxel downsampling is applied to the cropped cell point cloud to mitigate this issue. The impact of different downsampling levels (256, 512, 1024, 2048, 3072, 4096) on phenotype estimation is compared. To augment the point cloud data and leverage the RGB information embedded in each point cloud, data augmentation techniques such as random hue shifting, brightness adjustment, contrast modification and saturation variation are employed. Random Gaussian perturbation and rotation methods are utilized to expand the dataset for point clouds with 3D spatial attributes (x, y, z).

#### 3.2.2 Point cloud network architecture

**Fig. 2:**
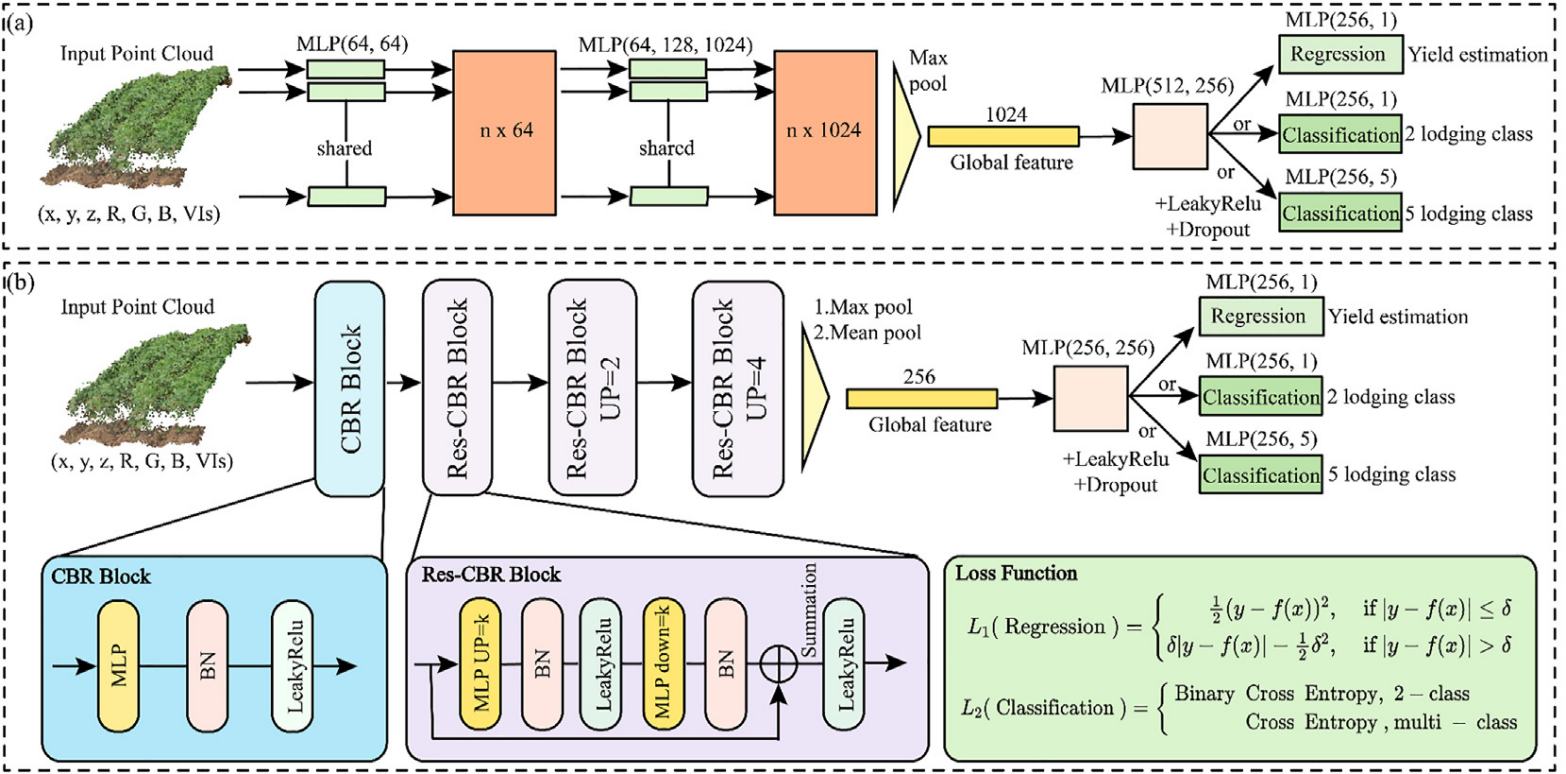
Deep Learning framework for point cloud: (a) SoyNet: The network architecture is based on PointNet-basic. The input point cloud contains information of (x, y, z, R, G, B, VIs), which is aggregated using the max pooling method after two MLP layers to obtain a global feature with 1024 dimensions. Yield estimation or lodging classification with 5 categories or lodging classification with 2 categories are finally performed after processing through two MLP layers. (b) SoyNet-Res: The input point cloud contains information of (x, y, z, R, G, B, VIs). The feature dimension is first increased using CBR Block and then either max pooling or mean pooling is used for feature aggregation after three Res-CBR blocks to obtain global features with 256 dimensions. The Loss Function for regression task and classification task can be seen in the loss function.

Two deep learning network architectures are established for point clouds, namely SoyNet and SoyNet-Res, and their effects and roles in phenotype estimation are compared (Fig. 2). Among them, SoyNet (Fig. 2a) adopts the PointNet-basic network while eliminating the T-Net layer with low contribution [46]. The point cloud data is first input into the network contains both spatial and spectral dimension information, which undergoes transformation through a 64 ​× ​64-dimensional multilayer perceptron (MLP) layer. The MLP layer with dimensions of 64 × ​128 ​× ​1024 is then input and passed through the max pooling to obtain the Global feature. It goes through a 512 ​× ​256 dimensional MLP layer with LeakyRelu activation function to prevent overfitting during model training. A Dropout layer is utilized with a dropout rate set at 60 ​%. Finally, the features are fed into three independent MLP layers for further processing. MLP layers with 256 ​× ​1 dimensional are employed for regression analysis of yield estimation. A single-layered MLP with dimensions of 256 ​× ​1 is used for binary classification task distinguishing lodging (greater than level 1) from non-lodging (level 1). MLP layers with dimensions of 256 ​× ​5 is employed for lodging at five-class discrimination.

SoyNet-Res (Fig. 2b) is established by combining PointNet and PointMLP [47] based on the residual structure in the point cloud network. The input consists of point clouds with spatial information and spectral dimension information, which are passed through a CBR block composed of MLP, Batch Normalization (BN), and LeakyRelu layers. Feature transformation is then performed using three Res-CBR block layers. The Res-CBR block transforms the point cloud features through dimension raising MLP (up ​= ​k, where k represents multiple) layer, BN layer, LeakyRelu layer, dimension reducing MLP (down ​= ​k) layer with BN layer. The original input features are directly summed with the transformed features to form the residual structure. Finally, the feature is entered into the LeakyRelu layer. The max pooling strategy and mean pooling strategy are compared for obtaining a 256-dimensional global feature through feature aggregation. Phenotype estimation and discrimination are finally performed using an architecture similar to Fig. 2a consisting of a 256 ​× ​256 dimensional MLP layer followed by LeakyRelu layer, Dropout layer and another MLP layer.

#### 3.2.3 Loss functions for point cloud deep learning

Yield estimation is a regression task, and the employed Loss function follows the Huber Loss [48] formulation (Eq. (1)), where y represents the measured phenotype value, x denotes the input data, f signifies the model prediction function, f(x) represents the model's predicted outcome given input x, and δ is a control coefficient set to 0.10. Lodging discrimination involves a classification task (Eq. (2)), with Binary Cross Entropy utilized for binary lodging discrimination and Cross Entropy employed for lodging at five-class discrimination. *N* represents the number of samples in a set of batches.

(1) $$\begin{array}{c} L_{1}\left(Regression\right)=\left\{ \begin{array}{c} {\frac{1}{2}\left(y-f\left(x\right)\right)}^{2},if\;\left|y-f\left(x\right)\right|\leq \delta \\ \delta \left|y-f\left(x\right)\right|-\frac{1}{2}\delta^{2},if\;\left|y-f\left(x\right)\right|>\delta \end{array}\right. \end{array}$$

(2) $$\begin{array}{c} L_{2}\left(Classification\right)=\left\{ \begin{array}{c} \frac{1}{N}\sum_{i=1}^{N}\begin{array}{c} (y_{i}\cdot \ln\left(f\left(x_{i}\right)\right)+\\ \left(1-y_{i}\right)\cdot \ln\left(1-f\left(x_{i}\right)\right)) \end{array},2-class\\ -\sum_{i=1}^{N}y_{i}\;\ln\left(f\left(x_{i}\right)\right),multi\;class \end{array}\right. \end{array}$$

### 3.3 Multi-task point cloud deep learning

#### 3.3.1 Network architecture of multi-task point cloud deep learning

Single-task deep learning (SDL) model is trained using independent target information and a separate loss function, which directs the model's attention towards the task of estimating the target phenotype. However, in multi-phenotype estimation tasks, there exist interdependencies among multiple phenotypic features. Therefore, compared to SDL, multi-task deep learning (MDL) can effectively enhance the generalization ability of the model and mitigate overfitting by leveraging mutual assistance from different target tasks [29,30,49].

**Fig. 3:**
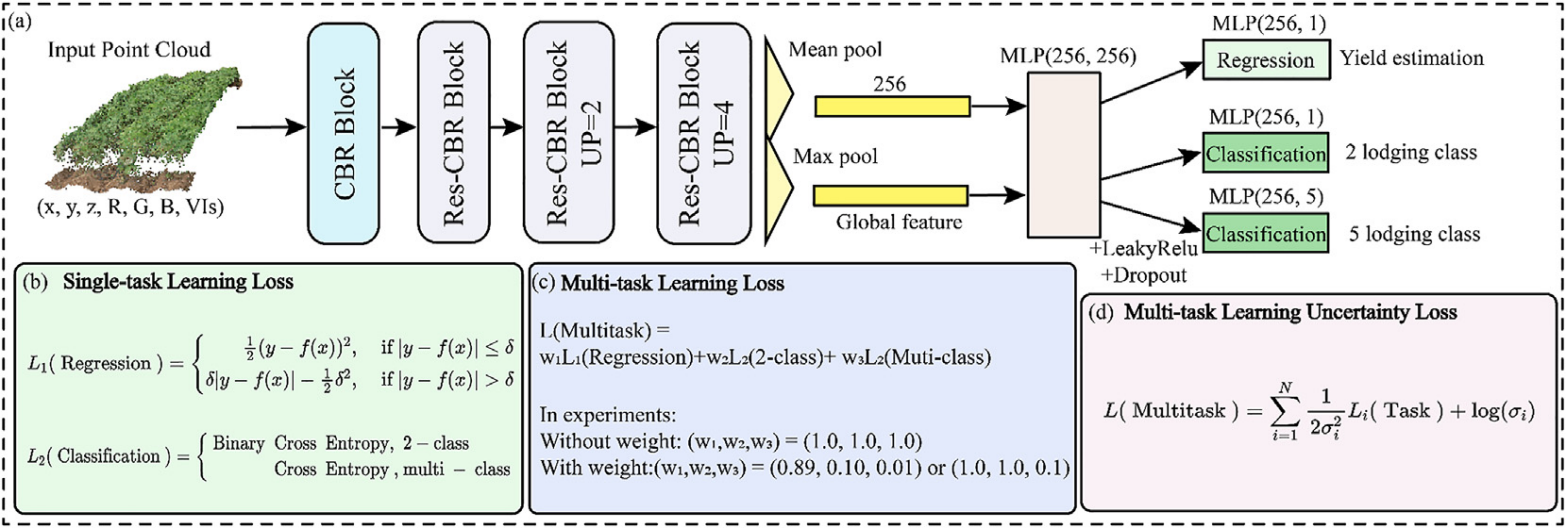
MDL framework for point cloud: (a) The network architecture for MDL involves inputting point cloud feature information and passing it through CBR Block and Res-CBR Block modules, as depicted in Fig. 2b. Feature aggregation is performed using mean pooling or max pooling to obtain 256-dimensional global features. Finally, MLP layers with LeakyReLU activation, Dropout regularization, and additional MLP layers are utilized for yield estimation or lodging at multi-class discrimination. (b) Loss function for SDL. (c) Loss function for MDL: a weighted combination of multiple single-task loss functions is employed. In the experiment, when unweighted is applied, it is set to (1.0, 1.0, 1.0). When weight is used, it can be set to either (0.89, 0.10, 0.01) or (1.0, 1.0, 0.1). (d) MDL(dynamic weight) loss function: the model autonomously assigns uncertainty weights to each task and combines them in the process of MDL.

In contrast to SDL, MDL involves interdependent tasks. Effective multi-objective training is achieved through weighted fusion of multiple loss functions. In the context of MDL (Fig. 3a), three tasks, namely yield estimation, lodging at five-class discrimination, and lodging at two-class discrimination, are simultaneously trained using three methods for fusing loss functions: unweighted fusion, weighted fusion, and homoscedastic uncertainty weight fusion [29]. The architecture of MDL (Fig. 3a) and SoyNet-Res (Fig. 2b) also employ CBR Block and Res-CBR Block for feature transformation. However, in the aggregation layer, mean pooling is used for yield estimation while max pooling is used for lodging discrimination. Common features are finally obtained through a 256 ​× ​256 dimensional MLP layer along with LeakyRelu layer and Dropout layer with a dropout rate of 60 ​%. Multi-task training utilizes an independent MLP layer.

#### 3.3.2 Loss functions of multi-task deep learning

The MDL function is formed by combining multiple SDL loss functions (Fig. 3b) through weighting, as illustrated in Eq. (3). The model assigns weights $w_{1}$, $w_{2}$, $w_{3}$ to ensure the aggregation of these SDL loss functions. In the experiment (Fig. 3c), the weighted fusion sets $w_{1}$, $w_{2}$, $w_{3}$ are set as 1.0, 1.0, and 1.0 respectively; whereas the weighted fusion learning sets as 0.89, 0.10, and 0.01 or alternatively as 1.0, 1.0, and 0.1 respectively to maintain a similar order of magnitude for the multi-task loss function. Multitasking performance heavily relies on the allocation of loss function weights to each task, making manual adjustment inefficient [50]. The optimal weight for each task depends on its magnitude and the level of task noise [29]. Multi-task uncertainty learning is a technique that allows a model to learn multiple tasks simultaneously while accounting for the uncertainty associated with each task [29,30]. By training a shared neural network, the loss function for each task is weighted by its uncertainty, ensuring that tasks with higher uncertainty receive less emphasis during training [49,50]. This leads to more robust and accurate predictions. This study shows that using multiple task loss functions with homoscedasticity uncertainty can effectively promote learning across diverse target tasks, known as MDL (dynamic weight). MDL (dynamic weight) loss function is shown in Eq. (4), where N is the number of multi-tasks and $\sigma_{i}$ is the standard deviation of the Gaussian distribution of the model for the ith task, which is also the noise used as the model. When $\sigma_{i}$ increases, the weight of the loss function of the corresponding task decreases. To make the model stable for MDL, we set the $\sigma_{i}$ of the model learning to $\ln\left(\sigma_{i}\right)$ and initialize it to 1.0.

(3) $$\begin{array}{c} L\left(Multitask\right)=w_{1}L_{1}\left(Regression\right)+w_{2}L_{2}\left(2\;class\right)+w_{3}L_{2}\left(muti\;class\right) \end{array}$$

(4) $$\begin{array}{c} L^{\prime }\left(Multitask\right)=\sum_{i=1}^{N}\frac{1}{2\sigma_{i}^{2}}L_{i}\left(Task\right)+\ln\left(\sigma_{i}\right) \end{array}$$

### 3.4 Model evaluation

#### 3.4.1 Evaluation of yield estimation

Soybean yield estimation accuracy is evaluated by Root mean squared error (RMSE), Correlation coefficient *r*, relative RMSE (rRMSE) and Mean absolute percentage error (MAPE), as shown in Eqs. (5), (6), (8)). Here, $y_{i}$ and $\hat{y_{i}}$ are the measured and estimated values of soybean yield, respectively. $\bar{y}$ is the average of the measured soybean yield, and n is the total number of samples in the test set.

(5) $$\begin{array}{c} RMSE=\sqrt{\frac{\sum_{i=1}^{n}{\left(y_{i}-\hat{y_{i}}\right)}^{2}}{n}} \end{array}$$

(6) $$\begin{array}{c} r=\sqrt[{2}]{1-\frac{\sum_{i=1}^{n}{\left(y_{i}-\hat{y_{i}}\right)}^{2}}{\sum_{i=1}^{n}{\left(y_{i}-\bar{y}\right)}^{2}}} \end{array}$$

(7) $$\begin{array}{c} rRMSE\%=\frac{RMSE}{\bar{y}}\times 100\% \end{array}$$

(8) $$\begin{array}{c} MAPE=\frac{100\%}{n}\sum_{i=1}^{n}\left|\frac{y_{i}-\hat{y_{i}}}{y_{i}}\right| \end{array}$$

#### 3.4.2 Evaluation of lodging discrimination

Accuracy, precision, recall, and F1-score are used to evaluate the discrimination accuracy of crop lodging and to measure the training quality of the model, as shown in Eqs. (9), (10), (11), (12)). TP is the number of true positive samples, FP is the number of false positive samples, and FN is the number of false negative samples. In addition, top-k accuracy was used to measure the lodging discrimination ability of the model. Top-k accuracy measures whether the top-k categories predicted by the model contain real category labels in a multi-classification problem.

(9) $$\begin{array}{c} Accuracy=\frac{TP+TN}{TP+TN+FP+FN} \end{array}$$

(10) $$\begin{array}{c} Precision=\frac{TP}{TP+FP} \end{array}$$

(11) $$\begin{array}{c} Recall=\frac{TP}{TP+FN} \end{array}$$

(12) $$\begin{array}{c} F1-score=2\cdot \frac{P\cdot R}{P+R} \end{array}$$

#### 3.4.3 Model validation strategy

From the 558 soybean plots, we surveyed and obtained the lodging severity and yield for each plot. To ensure the fairness of model validation, we used 5-fold cross-validation to assess model performance (Table S1). Due to the imbalance in the training set samples, we performed data augmentation. The training set and validation sets are independent, and each fold's validation set is also independent. After obtaining the results from all validation sets, we used the micro-average method to combine the prediction results from all classes and then calculated the overall performance metrics.

### 3.5 Model training environments and methods

The PaddlePaddle 2.4.0 framework is utilized in this study to construct the network with Python3.7. The GPU model of Tesla V100 is employed, while the CPU had 2 Cores and a memory capacity of 32 ​GB. For training the model, random values are used for initializing its parameters, and no transfer or training models are applied to the backbone section. The Momentum gradient descent algorithm is adopted with a momentum factor of 0.9, an initial learning rate of 0.02, and a learning rate decay strategy along with a weight decay set at 0.0001. The experiment underwent training for a total of 200 epochs with identical environmental settings and a batch size of 25 to ensure result comparability. In the statistical based phenotypic index estimation, H2O-AutoML [51] (3.46.0.1) is used to establish the automatic feature selection model. Ten models are used for comparison in the training process, and H2O-AutoML automatically selects the model with the best performance.

### 3.6 Spatial autocorrelation analysis

The presence of crop spatial dependencies within one or more fields has been observed [52]. Previous studies have typically assumed spatial independence as well as location invariance [53]. If spatial variation or autocorrelation is not considered, estimation models may lead to inaccuracy [54]. Therefore, it is meaningful to evaluate the effect of space on yield estimation and lodging discrimination by calculating the spatial autocorrelation of estimation errors [7]. Global Moran's I (MI) [55] is employed to assess the spatial distribution pattern of regression residuals in yield estimation models, aiming to examine the impacts of spatial variability induced by different varieties, planting densities, and other environmental factors. Global Moran's I value range from −1.0 to 1.0, indicating the presence of negative to positive spatial autocorrelation. Conversely, when the Global Moran's I values approach 0, it suggests a higher level of randomness and thus better model performance [55]. Z-score and p-value are statistical measures employed to assess the significance of the Moran index. If the p-value exceeds the predetermined significance level (P ​> ​0.05), the hypothesis is accepted with significant spatial randomness observed.

## 4 Result

### 4.1 Effect of the number of point clouds on the estimation

**Fig. 4:**
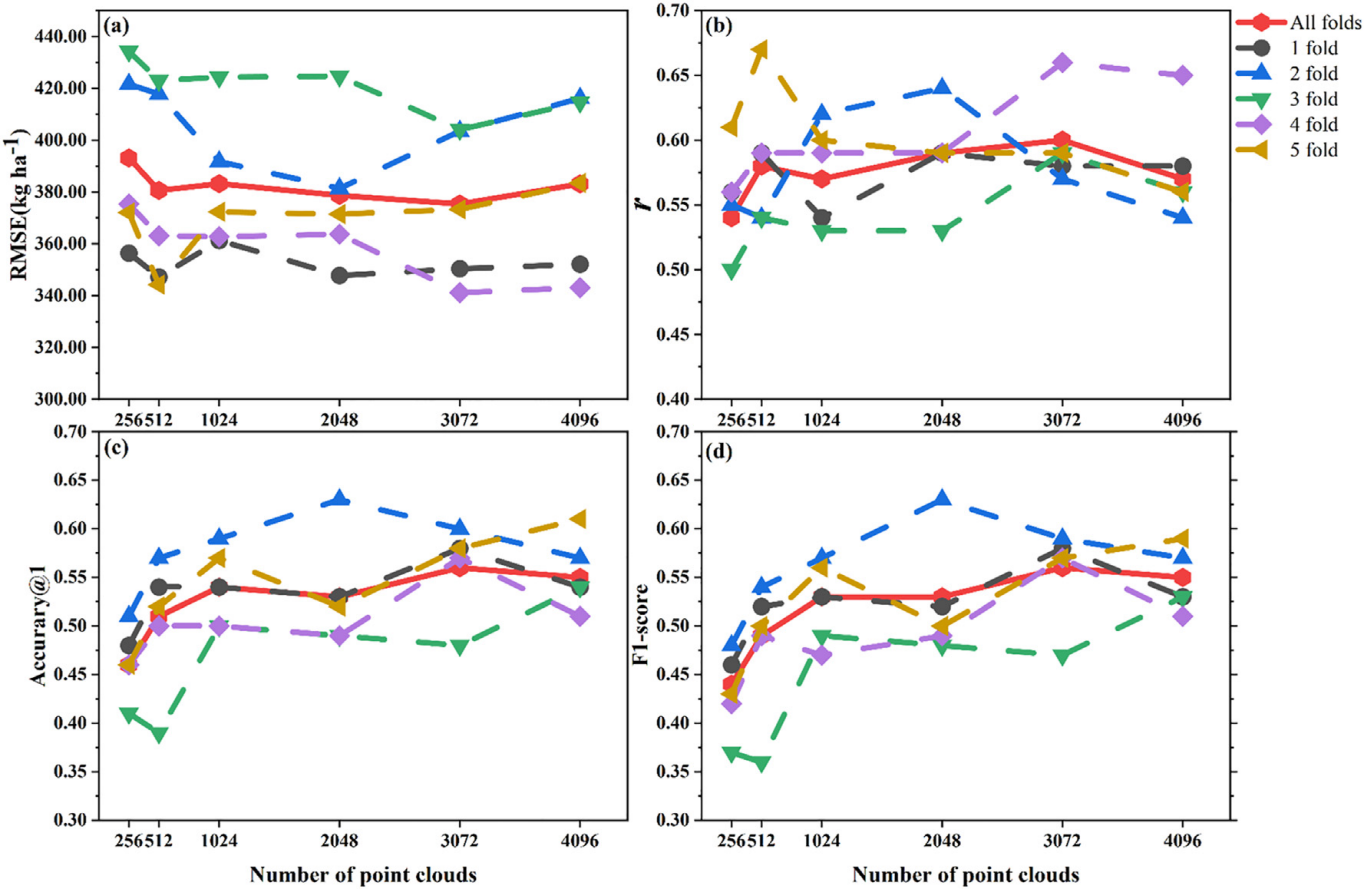
The results of yield estimation and lodging at five-class discrimination under different number of point clouds with the network structure of SoyNet at the soybean S7 growth stage. The evaluation index for yield estimation with RMSE (a) and *r* (b). The discriminant index of lodging at five-class discrimination with accuracy(c) and F1-score(d).

Although previous studies have typically used a point cloud network architecture with 1024 input points, further research is still needed to determine the optimal number of point clouds for plant phenotype estimation. Therefore, the optimal number of point clouds was investigated in the phenotype estimation task using the SoyNet (Fig. 2a) network architecture at the S7 of soybean growth stage in this study. The evaluation was conducted through 5-fold cross validation (Fig. 4). The dotted lines represent the outcomes of different folds, while the solid lines depict the comprehensive results obtained from 5-fold verification. A total of six groups of point clouds were employed for comparison purposes with the number of points as 256, 512, 1024, 2048, 3072, and 4096. As the number of point clouds increases, there is a continuous improvement in yield estimation accuracy. However, when reaching a count of 1024 points, the accuracy tends to approach saturation with no significant further enhancement (Fig. 4ab). The yield estimation results reached their optimum performance at a count of 3072 points (RMSE ​= ​375.37 ​ ​kg ​ha^−1^; *r* ​= ​0.60; Fig. 4ab). The result of lodging at five-class discrimination is illustrated in Fig. 4cd. When the number of point clouds increased from 1024 to 3072, the accuracy of lodging at five-class discrimination also reached the highest, and the accuracy top-1 reached a peak at 0.56 (Fig. 4). However, if the number of point clouds exceeds or equals to 4096, it will lead to increased network memory consumption which hampers deep learning training and results in a slight decrease in accuracy.

### 4.2 Influence of combining RGB and VIs information

A combined point cloud encompassing both structural dimensions and color attributes are then integrated with highly precise spatial information of point clouds and RGB data by CCO method (Fig. 2a). Three types of input for the SoyNet network architecture were compared (Table 3): solely spatial information of point cloud(x, y, z), fused spatial information of point cloud with RGB data (x, y, z, R, G, B), and fusion of spatial information of point cloud with RGB and VIs data (x, y, z, R, G, B, VIs). Compared to the usage of spatial information alone, incorporating RGB color information leads to improved accuracy in yield estimation as well as lodging discrimination across five classes. By utilizing spatial information with RGB and VIs data together, the best yield estimation accuracy was achieved with RMSE value of 352.82 ​kg ​ha^−1^ and correlation coefficient *r* of 0.66. The proposed method achieves an accuracy top-1 score of 0.62 ​at five-class discrimination.

**Table 3:** Accuracy of phenotype estimation for different combinations of input information.

Attribute type			Estimates of grain yield				Lodging at five-class discrimination			
x, y, z (Spat. Info)	RGB (Spec. Info)	VIs (Spec. Info)	RMSE (kg ha^−1^)	*r*	rRMSE%	MAPE	Accuracy top-1	Precision	Recall	F1 score
✓	×	×	375.37	0.60	14.83	0.13	0.56	0.56	0.56	0.56
✓	✓	×	364.86	0.62	14.41	0.13	0.59	0.59	0.59	0.59
✓	✓	✓	352.82	0.66	13.94	0.12	0.62	0.62	0.62	0.62

### 4.3 Effects of different network structures on phenotype estimation

The present study compared the effects of H2O-AutoML, SoyNet, and SoyNet-Res (max pooling) as well as SoyNet-Res (mean pooling) and PointNet++ [46] on yield estimation and lodging at five-class discrimination (Table 4). In comparison to the conventional method for phenotype estimation, the point cloud deep learning network demonstrates better accuracy, resulting in a reduction of RMSE by 6.58–10.38 ​kg ​ha^−1^ while increasing *r* by 0.02–0.03. The improvement in accuracy top-1 is particularly pronounced in the lodging discrimination task, with an increase ranging from 0.04 to 0.07. However, it should be noted that local feature extraction using PointNet++ [46] poses challenges in terms of convergence and yields lower accuracy.

**Table 4:** Comparison of the influence of network structure design on the results under different strategies.

Strategy	Estimates of grain yield				Lodging at five-class discrimination			
	RMSE (kg ha^−1^)	*r*	rRMSE%	MAPE	Accuracy top-1	Precision	Recall	F1 score
H2O-AutoML	363.20	0.63	14.36	0.13	0.55	0.55	0.55	0.55
SoyNet	352.82	0.66	13.94	0.12	0.62	0.62	0.62	0.62
SoyNet-Res (max pooling)	356.62	0.65	14.09	0.13	0.61	0.62	0.61	0.61
SoyNet-Res (mean pooling)	353.45	0.66	13.96	0.12	0.59	0.62	0.59	0.58
PointNet++	497.54	0.08	19.66	0.18	0.35	0.25	0.35	0.23

**Fig. 5:**
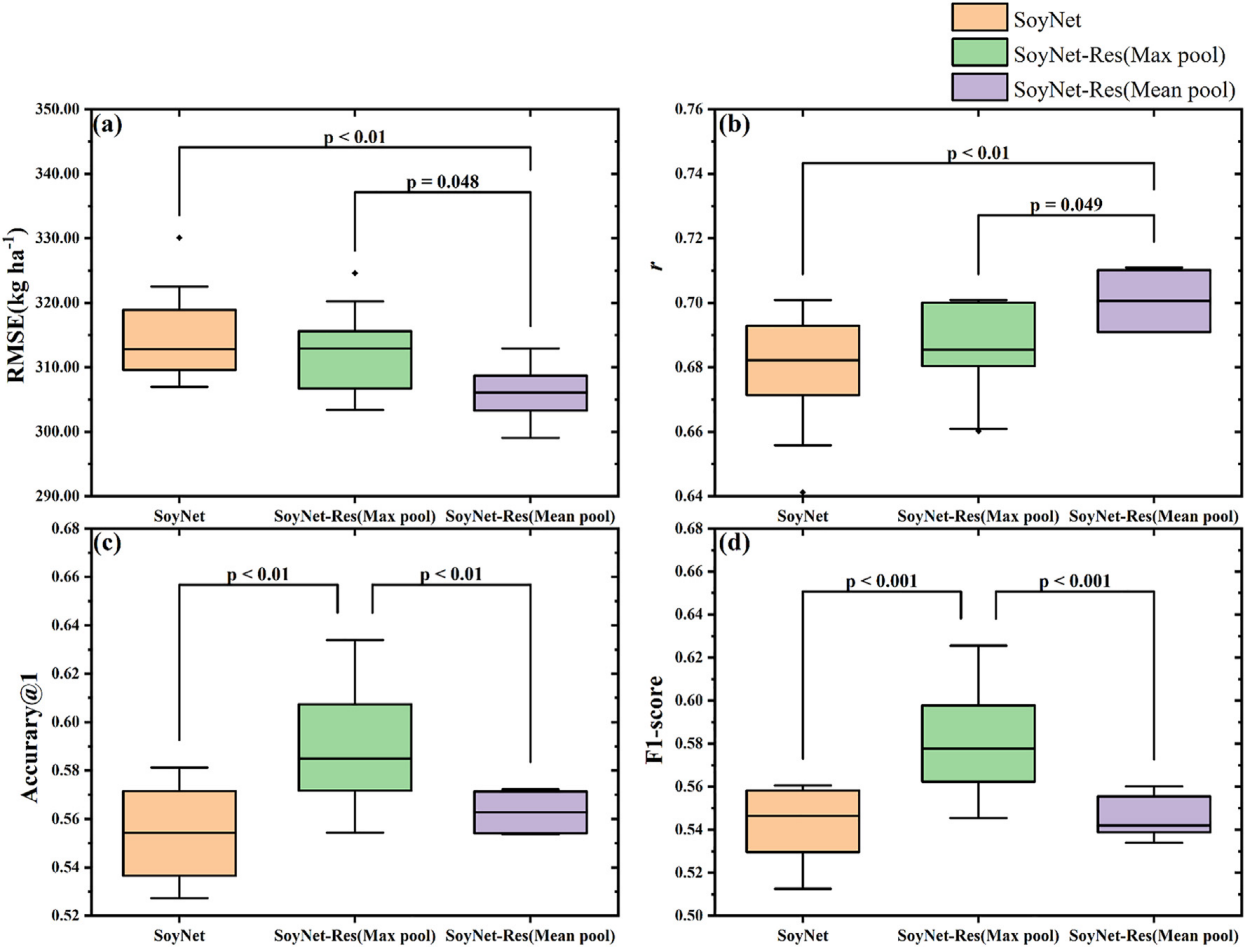
Comparisons of yield estimation and lodging discrimination for different network structures. The first fold was used as the training set for ten times of repetitive training to evaluate the training stability under different network structures. The evaluation index for yield estimation with RMSE (a) and *r* (b). The discriminant index of lodging at five-class discrimination with accuracy (c) and F1-score (d). Black dots represent outliers in the box plot.

When comparing SoyNet, SoyNet-Res (max pooling), and SoyNet-Res (mean pooling), the difference in accuracy was minimal (Table 4) between yield estimation and lodging discrimination. Therefore, stability experiments were conducted for three different strategies in this study, as illustrated in Fig. 5. To investigate the performance and applicability of the model across various strategies, all three methods underwent ten repetitions of training in the first fold, followed by a one-way analysis of variance (ANOVA) with honest significant difference (HSD) Tukey test (α ​= ​0.001). The results indicate that the SoyNet-Res network exhibits relatively higher accuracy and stability in the ten repeated training sessions compared to the SoyNet network (Fig. 5.), as evidenced by RMSE: 314.79 vs 305.71 ​kg ​ha^−1^, and standard deviation SD:7.34 vs 4.22. When comparing different aggregation methods, it is observed that the mean pooling method demonstrates better accuracy and stability in yield estimation (Fig. 5 ab), with RMSE: 305.71 vs 312.65 ​kg ​ha^−1^, SD:4.22 vs 6.63, p ​< ​0.048 indicating statistical significance. In terms of lodging at five-class discrimination task (Fig. 5cd), the max pooling method achieves higher accuracy and more significant differences, exhibiting accuracy top-1: 0.59 vs 0.56, SD:0 .01vs0 0.03, p ​≤ ​0 0.01.

### 4.4 Impact of multi-task assisted information fusion

**Fig. 6:**
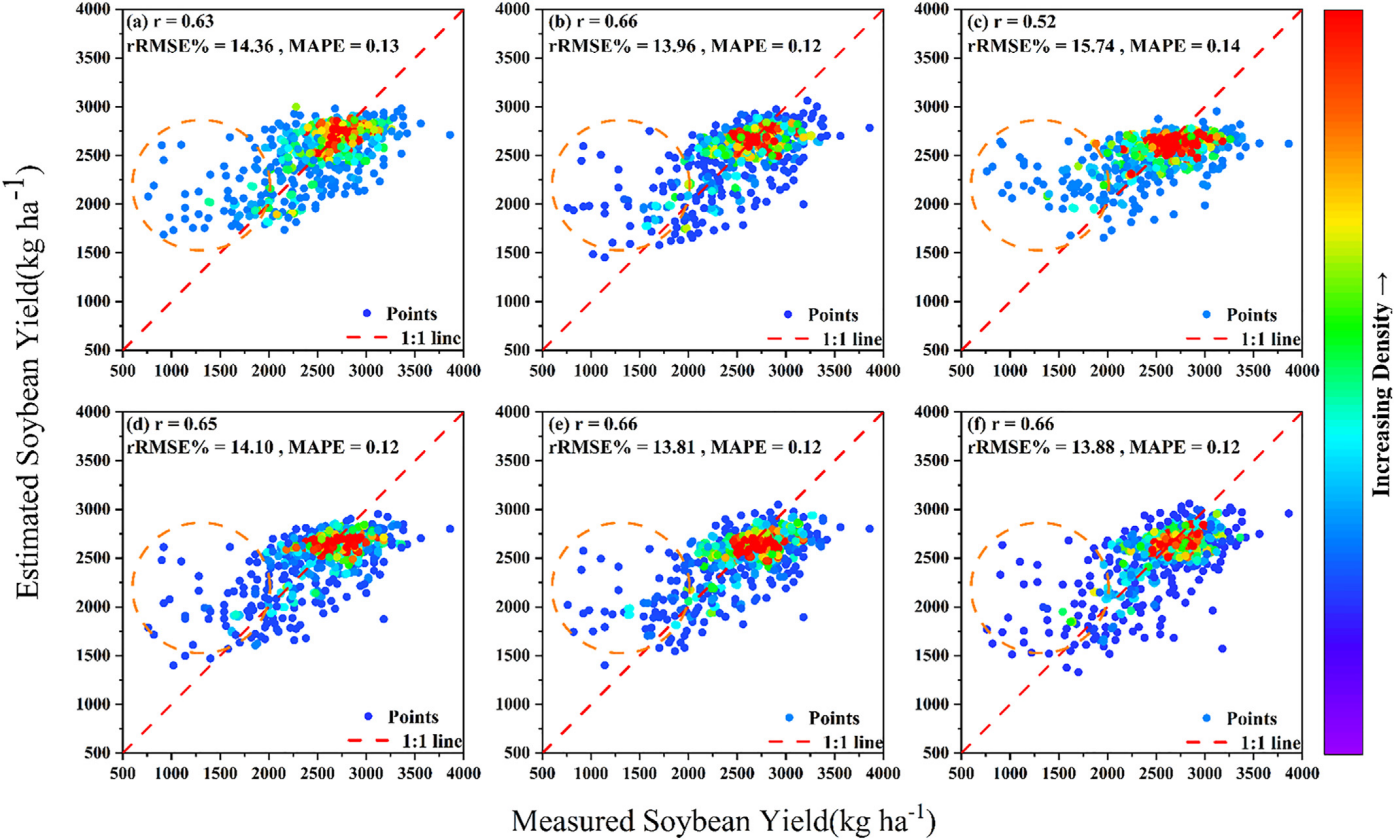
The yield estimations from different strategies are presented as scatter plots at the S7 growth stage. The orange circle area indicates estimated soybean yields that surpass the measured values. The legend on the right displays the density of scatter aggregation. The colors represent the density of the points. Red indicates areas with high point density, while blue indicates areas with low point density. (a) H2O-AutoML; (b)SDL-based SoyNet-Res (mean pooling); (c) MDL(unweighted); (d) MDL(1.0, 1.0, 0.1); (e) MDL(0.89, 0.10, 0.01); (f) MDL(dynamic weight).

The results of SDL and MDL are compared (Table 5) and a scatter plot of yield estimates is plotted (Fig. 6), and the top-k accuracy of lodging five-class discrimination is shown in Table 6. MDL-based (weighted) showed similar accuracy to SoyNet-Res for yield estimation, and achieved an improvement of 0.02–0.05 in accuracy for lodging discrimination tasks with both five-class and two-class discrimination. When comparing weighted and unweighted learning approaches in MDL, unweighted learning exhibited a decrease in accuracy for yield estimation task by approximately 0.13–0.14 of *r* and an increase in rRMSE by around 1.64–1.93 ​% (Fig. 6c). However, when comparing different weighted approaches within MDL (weighted 1 and 2), the difference in accuracy for yield estimation was minimal with RMSE of 349.45 vs356.95 ​kg ​ha^−1^ (Fig. 6d–f). For lodging at five-class discrimination, using MDL (dynamic weight) instead of fixed weight allowed for more effective MDL, and resulted in the highest classification accuracy@2 of 0.87 and accuracy top-3 of 0.97.

**Table 5:** Comparison of SDL and MDL for yield estimation and lodging classification.

Strategy	Weights			Estimates of grain yield					Lodging at five-class discrimination				Lodging at two-class discrimination			
	Grain yield	Lodging of 5	Lodging of 2	RMSE (kg ha^−1^)	RMAE (kg ha^−1^)	*r*	rRMSE	MAPE	Accuracy top-1	Precision	Recall	F1- score	Accuracy top-1	Precision	Recall	F1- score
SoyNet-Res (mean pooling)	1.00			353.45	265.76	0.66	13.96	0.12								
SoyNet-Res (max pooling)		1.00							0.61	0.62	0.61	0.61				
			1.00										0.88	0.88	0.88	0.88
MDL (unweighted)	1.00	1.00	1.00	398.43	298.62	0.52	15.74	0.14	0.63	0.64	0.63	0.63	0.91	0.90	0.91	0.90

MDL (weighted1)	1.00	0.10	1.00	356.95	272.74	0.65	14.10	0.12	0.64	0.64	0.64	0.64	0.91	0.91	0.91	0.91
MDL (weighted2)	0.89	0.01	0.10	349.45	263.90	0.66	13.81	0.12	0.64	0.64	0.64	0.64	0.91	0.90	0.91	0.90

MDL homoscedastic uncertainty weights	dynamic weight			351.43	262.00	0.66	13.88	0.12	0.66	0.66	0.66	0.66	0.91	0.91	0.91	0.91

Note: SDL (Single-task deep learning); MDL (Multi-task deep learning).

**Table 6:** Top-k accuracy for lodging 5-class discrimination.

Strategy	Accuracy of lodging 5-class discrimination			
	Top-1(@1)	Top-2(@2)	Top-3(@3)	Top-4(@4)
SoyNet-Res (max pooling)	0.61	0.86	0.96	0.99
MDL (unweighted)	0.63	0.85	0.97	0.99
MDL (weighted1)	0.64	0.86	0.97	0.99
MDL (weighted2)	0.64	0.86	0.96	0.98
MDL homoscedastic uncertainty weights	0.66	0.87	0.97	0.99

### 4.5 Effects of different growth stages on phenotypic estimation

**Fig. 7:**
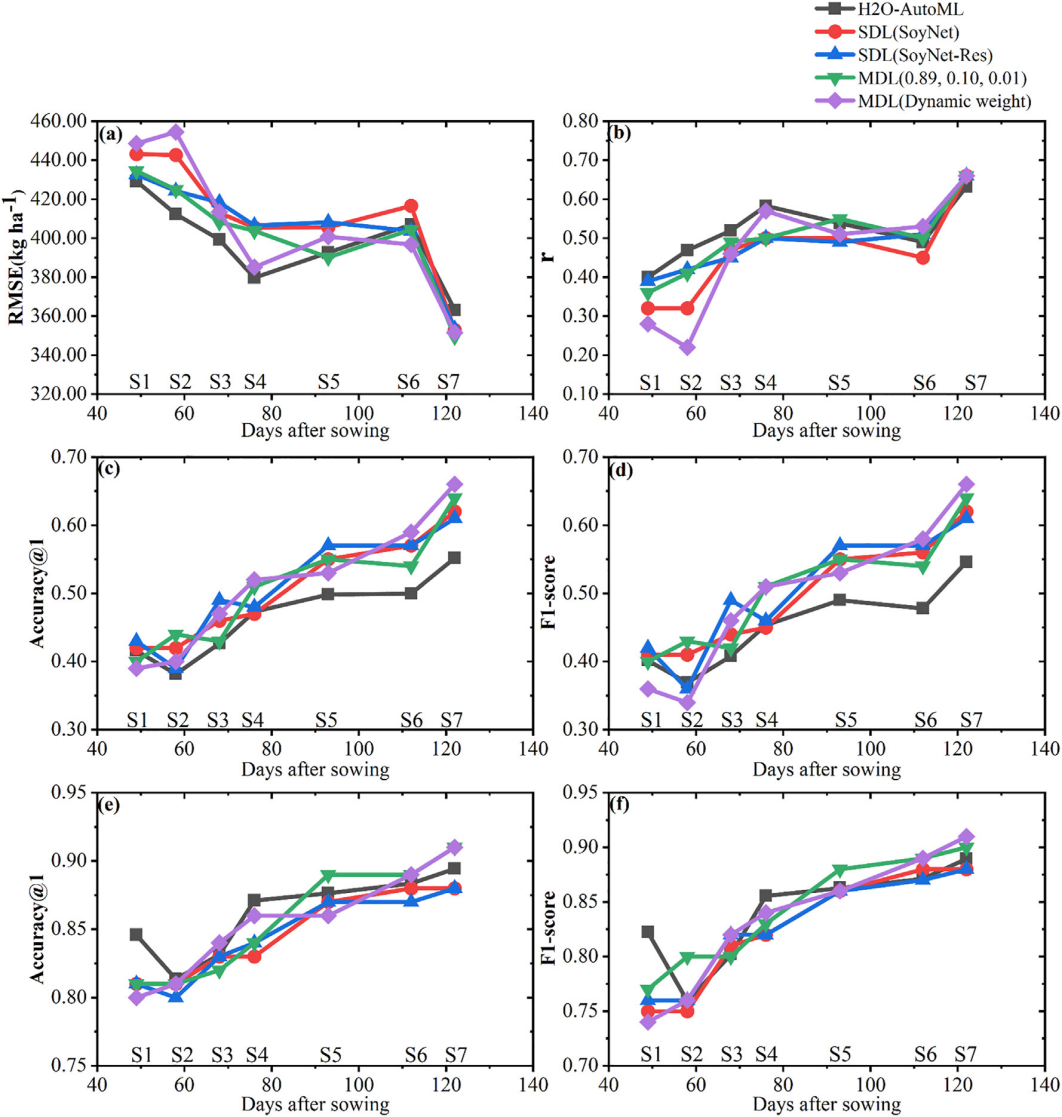
The evaluation of soybean yield estimation, lodging at five-class and two-class discrimination tasks at various growth stages with different methods. The evaluation index for yield estimation with RMSE (a) and *r* (b). The discriminant index of lodging at five-class discrimination with accuracy top-1 (c) and F1-score (d). The discriminant index of lodging at two-class discrimination with accuracy (e) and F1-score (f).

Influence of different growing stages on the accuracy of yield estimation and lodging discrimination was shown in Fig. 7. Different methods were compared in the study, such as H2O-AutoML with statistical based phenotypic index extraction method, SoyNet model, SoyNet-Res model, MDL (0.89, 0.10, 0.01), MDL (dynamic weight), for yield estimation, lodging at five-class and two-class discriminations. In the yield estimation task (Fig. 7ab), the RMSE values of different methods exhibited a pattern of initial decrease, followed by a slight increase and subsequent decrease. The optimal growth stage for soybean yield estimation was found to be at the S7 growth stage, with an RMSE value of 349.45 ​kg ​ha^−1^. The second highest RMSE value was observed at the S4 growth stage with value of 379.68 ​kg ​ha^−1^. In the lodging at five-class discrimination task (Fig. 7cd), there was a gradual increase in accuracy top-2 up to 0.87 as the soybean growth process advanced. However, the accuracy achieved by MDL method surpassed that of H2OAuto-ML at the growth stages S5, S6, and S7, highlighting its effectiveness in this context. Regarding lodging at two-class discrimination task, accuracy also improved gradually with increasing growth stages at maximum accuracy level of 0.91.

### 4.6 Spatial analysis of soybean phenotype estimation

Relative errors for the spatial autocorrelation of yield estimation and lodging discrimination at the S7 growth stage were calculated using SoyNet-Res, MDL weighted (0.89, 0.10, 0.01), and MDL (dynamic weight) (Table 7). In the yield estimation task, there is a weak spatial autocorrelation with a Moran's I value ranging from 0.107 to 0.127 due to the small difference in prediction accuracy. For lodging at five-class discrimination, the lowest error and spatial autocorrelation Moran's I ​= ​−0.037 are achieved by employing SDL with SoyNet-Res (max pooling) network structure. In lodging at two-class discrimination, the use of MDL (dynamic weighted) results in achieving the lowest spatial autocorrelation Moran's I ​= ​−0.060. Corresponding to these three strategies mentioned above, error spatial distribution maps for yield estimation(Fig. S1), lodging at five-class discrimination (Fig. S2), and lodging at two-class discrimination (Fig. S3) were generated (Supplemental Material2).

**Table 7:** Estimation result error for spatial autocorrelation calculation under different strategies.

Strategy	Phenotype task	Metrics		
		Moran's I	Z Score	P
H2O-AutoML	Grain yield	0.083	2.009	0.044
	Lodging at five-class discrimination	−0.002	0.204	0.837
	Lodging at two-class discrimination	0.177	4.091	0.001
SDL-based SoyNet-Res	Grain yield	0.110	2.649	0.008
	Lodging at five-class discrimination	−0.037	−0.793	0.428
	Lodging at two-class discrimination	0.021	0.525	0.600
MDL-based (0.89, 0.10, 0.01)	Grain yield	0.107	2.587	0.009
	Lodging at five-class discrimination	0.020	0.495	0.620
	Lodging at two-class discrimination	0.070	1.641	0.101
MDL-based (dynamic weight)	Grain yield	0.127	3.063	0.002
	Lodging at five-class discrimination	0.020	0.497	0.619
	Lodging at two-class discrimination	−0.060	−1.327	0.184

## 5 Discussion

### 5.1 The effect of network structure on phenotypic estimation

The proposed SoyNet model integrates RGB color and VIs information as input data, expanding the dimension from 3 to 13 in comparison with the PointNet-basic network structure [46]. To address the task of yield estimation, a new regression head is incorporated into the SoyNet model. The SoyNet-Res model replaces the MLP layer of SoyNet with the new CBR Block and Res-CBR block, thereby enhancing model convergence [56] and improving training stability (Fig. 5).

In terms of pooling methods, the max pooling, mean pooling, and Sum pool techniques are primarily employed to ensure rotation invariance of point clouds [57]. The experiment compares the efficacy of max pooling and mean pooling (Fig. 5). There was no significant difference in accuracy when utilizing either the max pooling or mean pooling method for yield estimation or lodging discrimination purposes. However, in comparison of the results from ten repeated experiments, the mean pooling method demonstrates better accuracy and stability (RMSE: 305.71 vs 312.65 ​kg ​ha^−1^, SD:4.22 vs 6.63) in yield estimation (Fig. 5ab), making it more suitable for regression tasks of the related phenotypic analysis. The max pooling method achieves higher accuracy and exhibits a more significant difference (Accuracy top-1: 0.59 vs 0.56, SD:0.01 vs 0.03, p ​< ​0.01) in the lodging discrimination task (Fig. 5cd), making it more appropriates for tasks in the classification such as lodging discrimination and point cloud classification.

The accuracy of phenotype estimation is significantly influenced by the growth stages of soybean. Bai [58] utilized UAV RGB images to collect data at 17 times throughout the soybean growing season, revealing that the flowering stage was most suitable for soybean yield estimation (R^2^ ​= ​0.50, rRMSE% ​= ​43.71 ​%). Maimaitijiang [7] conducted yield estimation for three soybean varieties at the first pod stage (R3 stage) through UAV multi-modal data fusion, resulting in an R^2^ value of 0.72 and rRMSE% of 15.90 ​%. In this study, we evaluated the soybean yield estimation at seven different growth stages (Fig. 7). The results demonstrated that soybean yield estimation achieved a relatively high accuracy (RMSE: 379.68 ​kg ​ha^−1^) during the S4 growth stage, which was consistent with the previous studies [6,59]. However, the canopy structure became more complex and the spectrum saturation increase [60] between 76 and 112 days after sowing, leading to a gradual decrease in yield estimation accuracy. Considering the diversity of soybean varieties, different developmental conditions are observed during the process of yield estimation, making it challenging to determine the optimal stage for estimating yields with the consideration of variety diversity [17].

### 5.2 The significance of multi-task deep learning

The approach of MDL involves simultaneous learning of multiple tasks by sharing model parameters, which offers the advantages of enhancing data efficiency, mitigating overfitting through shared representations, and leveraging auxiliary information for rapid learning [31,50,61]. By incorporating parameters into phenotypic estimation, multi-task learning identifies potential related features of yield and lodging to enhance learning efficiency and accuracy, resulting in a slight improvement in the discrimination of lodging at five-class (Table 5).

Manual weight setting is an inefficient and challenging task, as the scale of loss varies significantly across different tasks [29]. This can result in one task dominating the loss function, rendering the losses of other tasks irrelevant to the learning process of network sharing layer [62,63]. Therefore, it is imperative to automatically set weights for multiple tasks. Although MDL (dynamic weight) achieves similar results to manual weights in yield estimation (RMSE:356.95 VS 351.43 ​kg ​ha^−1^), it eliminates the need for adjusting weight parameters and achieves a better F1-score ​= ​0.66 in lodging at five-class discrimination.

### 5.3 Applicability and limitations of point cloud deep learning

In previous studies, VIs and CC were typically derived from 2D data such as multispectral and hyperspectral data to estimate phenotypic parameters [6,64]. Canopy 3D models were primarily obtained from DSM or point cloud, enabling the extraction of plant height [6,7,24,64], along with the utilization of 3D voxel index (3DVI) and 3D profile index (3DPI) [25], quantile percentage analysis based on different height categories, and other related parameters. However, according to the statistical characteristics, the crucial structural information embedded in the 3D data is easy to be lost [28]. Li et al. [65] proposed a method to reduce point cloud data to 2D images, but there was still information loss caused by dimensionality reduction, and the problem of modeling directly from data was not solved. Therefore, a deep learning approach was proposed for direct modeling and processing of soybean point cloud data.

VIs are constructed by utilizing the RGB data values attached to the point cloud, enabling the model to simultaneously integrate spatial structure and color information. Compared to solely relying on spatial information of the point cloud (Table 3), integration with RGB color information and VIs yields higher accuracy for yield estimation of RMSE ​= ​352.82 ​ ​kg ​ha^−1^ and *r* ​= ​0.66. In terms of lodging classification, the accuracy top-1 achieves 0.60. The results further demonstrate that the deep learning network model based on point cloud effectively learns and fuses multi-source information, thereby enhancing phenotypic estimation accuracy.

The lack of interpretability in deep learning models, compared to statistical based phenotypic index, is considered a potential drawback. Deep learning can directly capture and learn intricate information from the data, and enhance model accuracy and eliminating the need for hyperparameter adjustment. However, challenges are posed in extracting meaningful insights [28]. To comprehend deep learning better, ongoing technical research will need to be relied on by further interpreting and visualizing deep learning models [66,67]. Multi-year experiments will also be further considered involving various crop types to validate the applicability and reliability of point cloud deep learning.

## 6 Conclusion

A lightweight unmanned aerial vehicle was employed in current study to integrate with the cross-circling oblique route to acquire high-precision soybean point clouds. The SoyNet and SoyNet-Res deep learning models for yield estimation and lodging discrimination were further established to utilize the acquired point clouds. The potential of MDL approach was further assessed for phenotype trait estimation. The key findings are summarized as follows.

(1) In estimating phenotypic traits, the SoyNet with deep learning achieved optimal yield estimation and lodging discrimination accuracy (RMSE ​= ​375.37 ​kg ​ha^−1^, *r* ​= ​0.60, F1-score ​= ​0.56) when using 3072-point clouds at S7 growth stage. The integration of color and VIs calculated with RGB resulted in improved prediction accuracy compared to using spatial structure information alone.
(2) The SoyNet-Res exhibited better accuracy compared to machine learning models. To enhance stability in yield estimation, the mean pooling method was employed for the SoyNet-Res model. In contrast, the max pooling method was utilized for the lodging discrimination task to achieve higher accuracy.
(3) Compared to SDL, MDL effectively capitalized on the correlations between different phenotypic tasks. In the case of MDL at soybean S7 growth stage, when adopting MDL (Dynamic weighted), the accuracy of yield estimation reaches its peak at *r* ​= ​0.66, while the Moran's I of the estimation error is 0.127, indicating a weak spatial autocorrelation. For lodging at five-class discrimination, the accuracy top-2 and Moran's I were 0.87 and 0.020, respectively. Specifically, lodging at two-class discrimination was 0.91 (F1-score) and −0.060 (Moran's I).

## Author contributions

Longyu Zhou: Writing – review & editing, Writing – original draft, Visualization, Validation, Software, Methodology, Formal analysis, Data curation, Conceptualization. Dezhi Han: Writing – review & editing, Validation, Data curation. Guangyao Sun: Formal analysis, Data curation, Conceptualization. Liu Yaling: Data curation. Xiaofei Yan: Formal analysis, Data curation, Conceptualization. Hongchang Jia: Resources, Data curation. Long Yan: Formal analysis, Resources. Puyu Feng: Conceptualization, Revision. Yinghui Li: Resources. Lijuan Qiu: Validation, Resources. Yuntao Ma: Supervision, Resources, Funding acquisition, Formal analysis, Modification and Revision.

## Data availability

The code and data mentioned in the article can be downloaded from https://gitlab.com/zlyzly28/plant-phenomics.

## Declaration of competing interest

The authors declare that they have no known competing financial interests or personal relationships that could have appeared to influence the work reported in this paper.
