# Supplementary material for: Soybean yield estimation and lodging discrimination based on lightweight UAV and point cloud deep learning
Source: Plant Phenomics. 2025 Mar 20;7(2):100028. doi: 10.1016/j.plaphe.2025.100028 (PMC12710009; doi:10.1016/j.plaphe.2025.100028)
Supplement: Multimedia component 1 [file mmc1.docx]

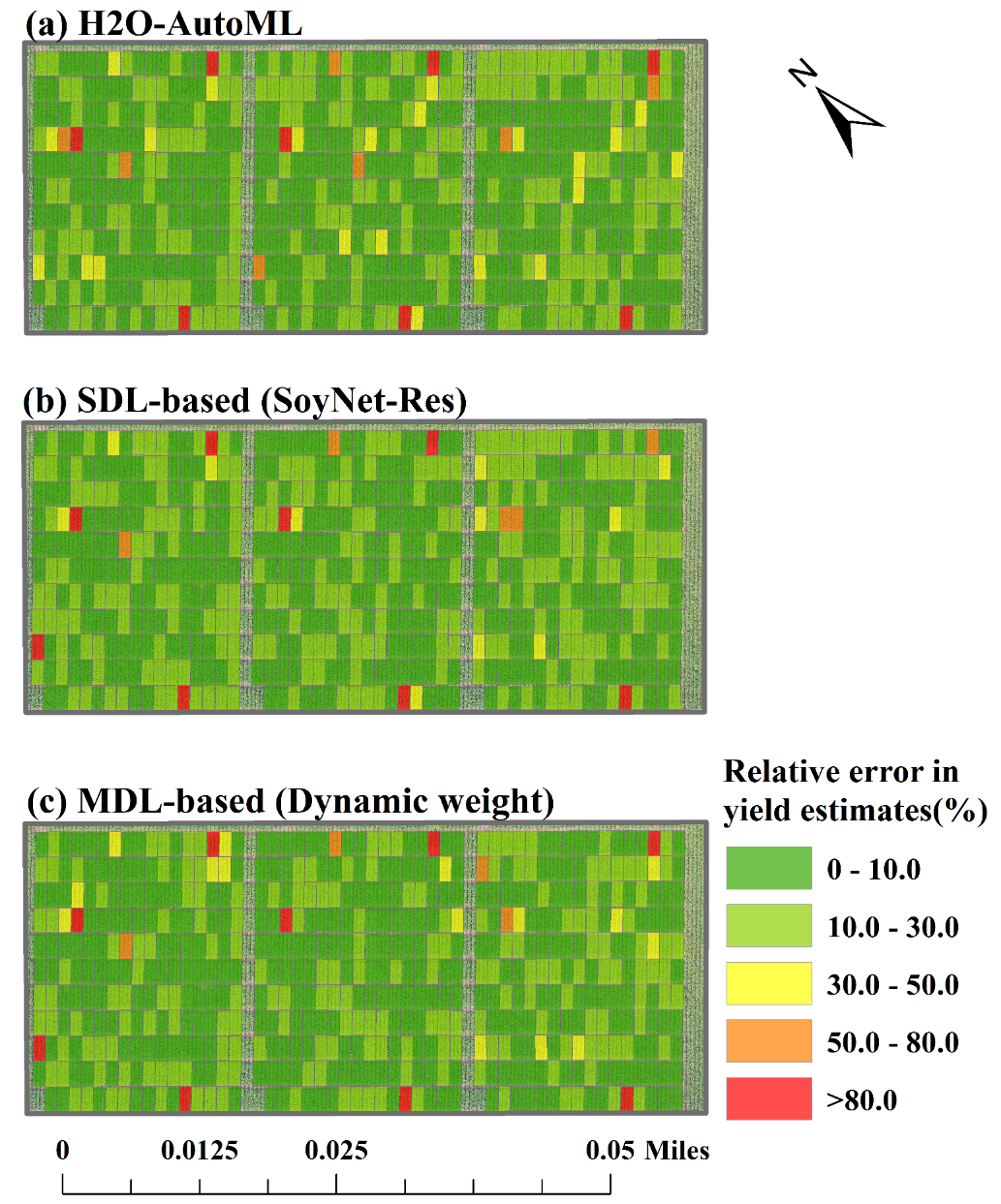


Fig. S1 The spatial distribution of relative errors for yield estimation calculated at the S7 growth stage. (a) H2O-AutoML. (b) SDL-based SoyNet-Res (mean pooling). (c) MDL (dynamic weight).


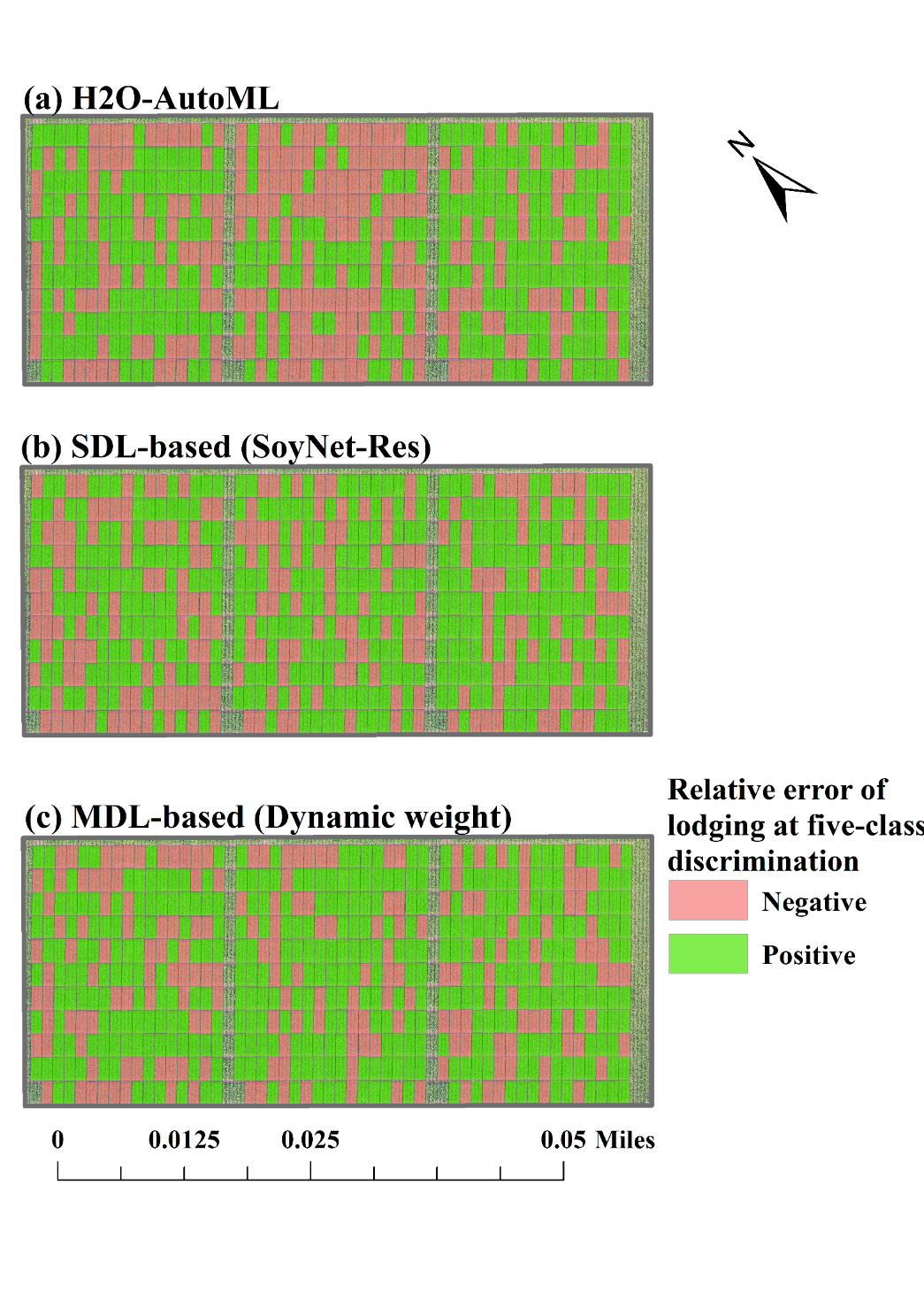


Fig. S2 The spatial distribution of relative errors for lodging at five-class discrimination observed at the S7 growth stage. (a) H2O-AutoML. (b) SDL-based SoyNet-Res (max pooling). (c) MDL (dynamic weight).


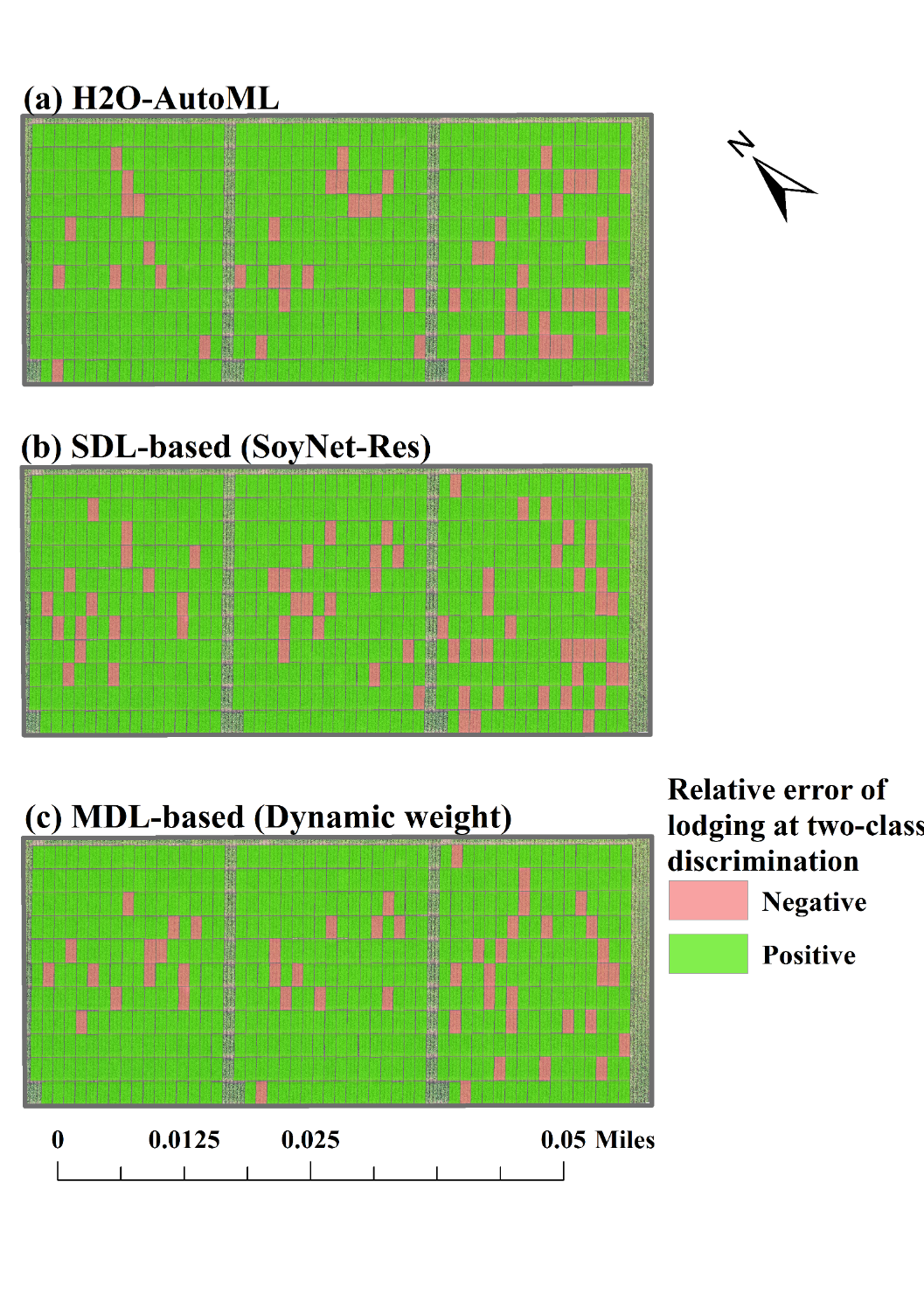


Fig. S3 The spatial distribution of relative errors for lodging at two-class discrimination observed at the S7 growth stage. (a) H2O-AutoML. (b) SDL-based SoyNet-Res (max pooling). (c) MDL (dynamic weight).
