# Supplementary material for: Soybean yield estimation and lodging discrimination based on lightweight UAV and point cloud deep learning
Source: Plant Phenomics. 2025 Mar 20;7(2):100028. doi: 10.1016/j.plaphe.2025.100028 (PMC12710009; doi:10.1016/j.plaphe.2025.100028)
Supplement: Multimedia component 2 [file mmc2.docx]

Table S1 Partitioning results of the data set

| Fold | Training set | Validation set | Data enhanced training set | Validation set |
| --- | --- | --- | --- | --- |
| 1-fold | 446 | 112 | 5225 | 112 |
| 2-fold | 446 | 112 | 5225 | 112 |
| 3-fold | 446 | 112 | 5225 | 112 |
| 4-fold | 446 | 112 | 5225 | 112 |
| 5-fold | 448 | 110 | 5225 | 110 |
